# Prediction and Observation of Post-Admission Hematoma Expansion in Patients with Intracerebral Hemorrhage

**DOI:** 10.3389/fneur.2014.00186

**Published:** 2014-09-29

**Authors:** Christian Ovesen, Inger Havsteen, Sverre Rosenbaum, Hanne Christensen

**Affiliations:** ^1^Department of Neurology, Bispebjerg Hospital, University of Copenhagen, Copenhagen, Denmark; ^2^Department of Radiology, Bispebjerg Hospital, University of Copenhagen, Copenhagen, Denmark

**Keywords:** intracranial hemorrhage, cerebral hemorrhage, cerebral angiography, X-ray computed tomography, transcranial ultrasonography

## Abstract

Post-admission hematoma expansion in patients with intracerebral hemorrhage (ICH) comprises a simultaneous major clinical problem and a possible target for medical intervention. In any case, the ability to predict and observe hematoma expansion is of great clinical importance. We review radiological concepts in predicting and observing post-admission hematoma expansion. Hematoma expansion can be observed within the first 24 h after symptom onset, but predominantly occurs in the early hours. Thus capturing markers of on-going bleeding on imaging techniques could predict hematoma expansion. The spot sign observed on computed tomography angiography is believed to represent on-going bleeding and is to date the most well investigated and reliable radiological predictor of hematoma expansion as well as functional outcome and mortality. On non-contrast CT, the presence of foci of hypoattenuation within the hematoma along with the hematoma-size is reported to be predictive of hematoma expansion and outcome. Because patients tend to arrive earlier to the hospital, a larger fraction of acute ICH-patients must be expected to undergo hematoma expansion. This renders observation and radiological follow-up investigations increasingly relevant. Transcranial duplex sonography has in recent years proven to be able to estimate hematoma volume with good precision and could be a valuable tool in bedside serial observation of acute ICH-patients. Future studies will elucidate, if better prediction and observation of post-admission hematoma expansion can help select patients, who will benefit from hemostatic treatment.

## Background

Early intervention is today a growing concept in the treatment of stroke patients. Patients arrive in the hospital early after the onset of stroke symptoms as a consequence of treatment options in ischemic stroke. Due to the ultra-early work-up, patients with intracerebral hemorrhages (ICH) will often be diagnosed quickly after symptom onset. ICH presents in general as devastating strokes, and patients are often prone to poor outcome. This is due to the stroke *per se* and the lack of treatment concepts proven to be effective in randomized clinical trials. Recently, both surgical intervention (1) and hemostatic therapy (2) have failed to improve the functional outcome.

As ICH is diagnosed increasingly early, post-admission hema- toma expansion will become a more frequent observation. Post-admission hematoma expansion contributes to the clinical instability of the patients, but at the same time it might be a promising target for interventions to limit final hematoma volume, save brain tissue – and improve functional outcome. Thus, being able to predict and dynamically observe post-admission hematoma expansion is of paramount clinical importance. In recent years, several radiological concepts to predict post-admission hematoma expansion have been identified. In this review, we summarize radiological predictors of hematoma expansion in patients with ICH. We further review new imaging concepts in dynamical observation of post-admission hematoma expansion.

## Post-Admission Hematoma Expansion

Post-admission hematoma expansion is defined as enlargement of the hematoma volume between the admission imaging procedure and a later imaging procedure. A variety of definitions of significant hematoma expansion have been proposed based on both relative (e.g., 33%) and absolute (e.g., 6 or 12.5 mL) hematoma volume increase. No convincing evidence exists as to which of these definitions best discriminate patients prone to poor functional outcome and all definitions yield equally low sensitivity (3). However, a trend might exist toward definitions based on absolute expansion being slightly more clinically meaningful in terms of outcome compared to definitions based on relative expansion (3).

**Figure 1:**
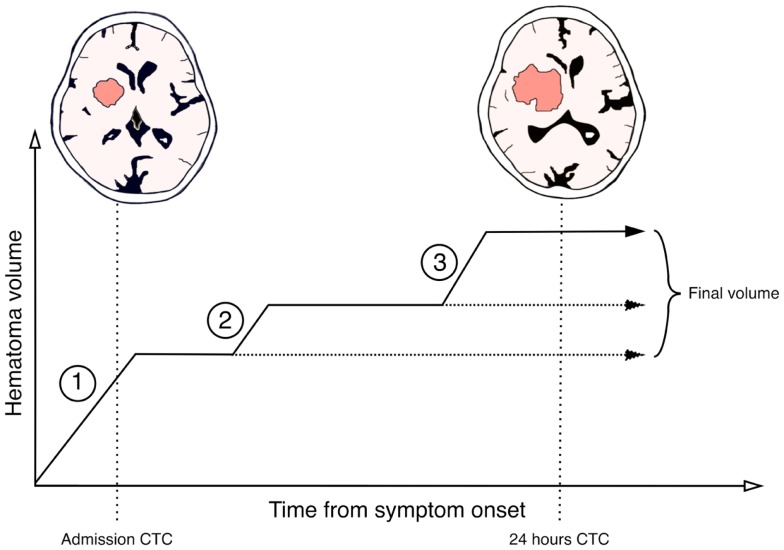
**Suggested schematic model for a step-wise hematoma expansion-profile in intracerebral hemorrhages**. Phase 1 represents the initial active bleeding-period most likely caused by the originally ruptured vessel. If patients are admitted within this phase an active hemorrhage will still be occurring post-admission and instant expansion can be observed. After phase 1 a plateau-phase might exist in which the hematoma volume is constant or active bleeding occurs slowly. In some patients, this phase might represent the final hematoma volume (horizontal stippled line). Phase 2 represents a secondary bleeding-period. This is likely due to secondary vessel rupture caused by pressure and ischemia on adjacent tissue structures or by re-bleeding from the originally ruptured vessel. Some patients might have multiple secondary bleeding-periods and some even late (phase 3). Between these periods are interpolated plateau-phases with relative constant hematoma volume or slow active bleeding. This results in the final hematoma volume observed on follow-up imaging.

Studies to determine the time interval of which post-admission hematoma expansion occur have been limited by the fact that serial radiological examinations in order to estimate the hematoma volume are hard to perform in an unstable patient-group and would potentially expose the patients to unacceptable amounts of ionizing radiation. Consequently, most published studies are limited by few prospective measurements or imprecise retrospective methodology. Studies indicate that post-admission hematoma expansion can be observed in up to 40% of patients admitted in an acute fashion (3). In a prospective study conducted on patients admitted within 3 h after symptom onset Brott et al. reported that 26% suffered hematoma expansion within the first hour after admission and additional 12% suffered expansion within the following 23 h (4). Ovesen et al. reported in another prospective study that active expansion could be observed within the first 8 h after symptom onset in the group of patients, who on 24 h follow-up CT-scan presented a final clinically relevant hematoma expansion (>12.5 mL) (5). Even though the vast majority of the hematoma expansion is observed to occur in the first hours after stroke onset, evidence also indicates that it is possible for hematoma expansion to take place later on and up to 24 h (6, 7). This supports the hypothesis that hematoma expansion (Figure 1) is a dynamic process with intermitting bleeding episodes caused by not only the originally ruptured vessel, but also by secondary rupture of adjacent vessels exposed to pressure and ischemia inflicted by the hematoma mass-effect (8). The secondary vessel rupture in the rim of the hematoma might explain that hematoma expansion does not happen symmetrically around the surface of the hematoma, but is located non-uniformly (9). Further, the secondary vessel rupture and intermitting bleeding episodes might also be the cause of the apparent plateau-phases with constant hematoma volume between active expansion episodes (5).

Post-admission hematoma expansion has been independently associated with early neurological deterioration (4, 10), poor functional outcome, and mortality (11, 12). An almost linear association between hematoma expansion and the probability of poor outcome has been reported – with 1 mL additional expansion yielding a 5% increased risk of death and dependency (12).

## Computed Tomography Angiography

Post-admission hematoma expansion occurs due to leakage of blood into an existing hematoma cavity. As previously discussed, this process represents an on-going or intermittingly on-going active bleeding process. Consequently, the ability to visualize this process of on-going bleeding could provide us with excellent prediction of hematoma expansion. It is an alluring thought that this active bleeding process could be visualized using radiographic contrast-agents, because leakage from surrounding vessels would be represented by pooling of contrast-material inside the hematoma. The concept of contrast-leakage into the hematoma in acute ICH-patients undergoing angiography dates back more than 40 years (13). It was originally observed that patients arriving earlier in the hospital more often demonstrated contrast-leakage. In recent years, much attention has been placed on the possibility of visualizing contrast-leakage using computed tomography angiography (CTA).

### Contrast-leakage on CT-imaging

**Figure 2:**
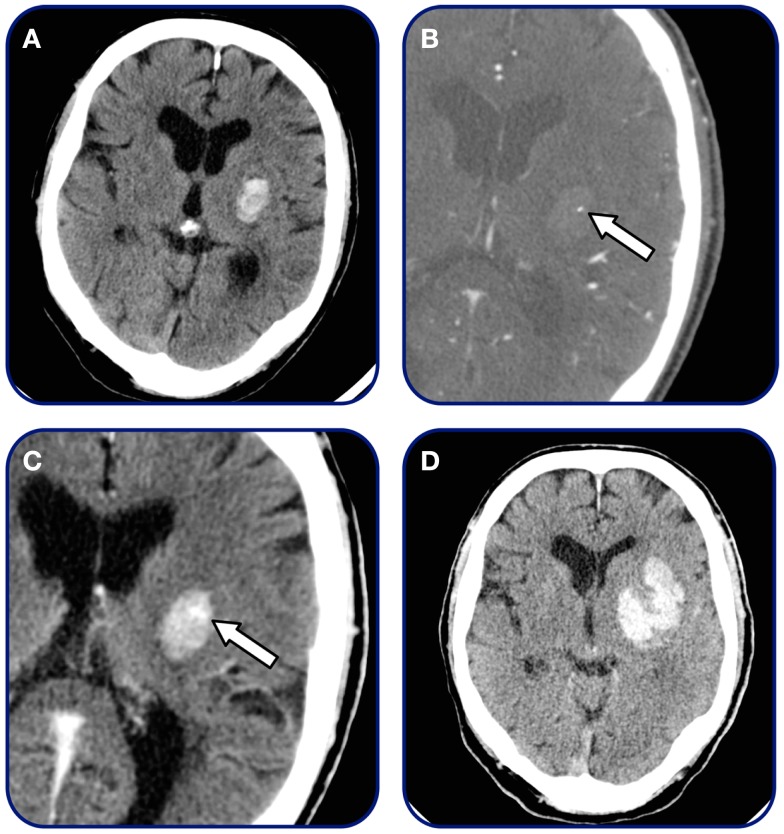
**Spot sign and contrast extravasation**. Patient admitted within 2 h after symptom onset. **(A)** Initial non-contrast CT demonstrating small basal-ganglia hemorrhage, **(B)** CTA source image demonstrating single spot sign (arrow), **(C)** 3-min post-contrast CT-imaging demonstrating contrast extravasation (arrow), and **(D)** 24 h follow-up CT revealing final hematoma volume.

Traditionally, two interrelated marker of contrast-leakage have been defined: the spot sign on CTA source images or maximum intensity projections and pooling of contrast (contrast extravasation) in the hematoma (Figure 2).

**Figure 3:**
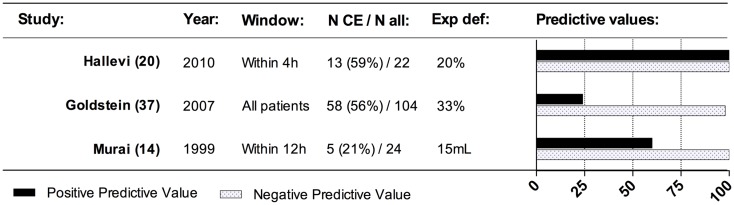
**Effect of contrast extravasation on hematoma expansion**. This figure presents the predictive values across studies for contrast extravasation to predict hematoma expansion. The predictive values indicated the independent effect of contrast extravasation and not the effect of contrast extravasation in addition to spot sign – please see text. N CE, number of patients with contrast extravasation; N all, total number of patients in the population; exp def, definition of hematoma expansion.

One of the first studies on contrast extravasation in patients with spontaneous ICH using CTA was presented by Murai et al. (14) in 1999. The study looked at extravasation of contrast on 3D-CTA images and concluded that extravasation was linked to hematoma expansion. Other studies have later supported this claim, and excellent negative predictive value in predicting hematoma expansion is indicated across studies performed (Figure 3). The concept of contrast extravasation in general refers to all manners of contrast-leakage into the hematoma. However, in more recent studies, the concept of contrast extravasation refers only to pooling of contrast in the hematoma on post-contrast CT-imaging following CTA (15). In this review, contrast-leakage will be used as an umbrella term for both contrast extravasation and the spot sign.

Wada et al. (16) was the first to describe small enhancing foci on CTA source images – the spot sign – and its ability to predict hematoma expansion. Ever since, many other studies have focused on the spot sign as a promising biomarker to predict hematoma expansion. Among studies, slightly different definitions of the spot sign have been used, however, many studies use definitions similar to the one proposed by Wada et al: “one or more, 1–2 mm foci of enhancement within the hematoma on CTA source images” or as proposed by Delgado Almandoz et al. (17): (i) 1 ≥ focus of contrast pooling within the ICH; (ii) with an attenuation ≥120 Hounsfield units; (iii) discontinuous from normal or abnormal vasculature adjacent to the ICH; and (iv) of any size and morphology.

**Figure 4:**
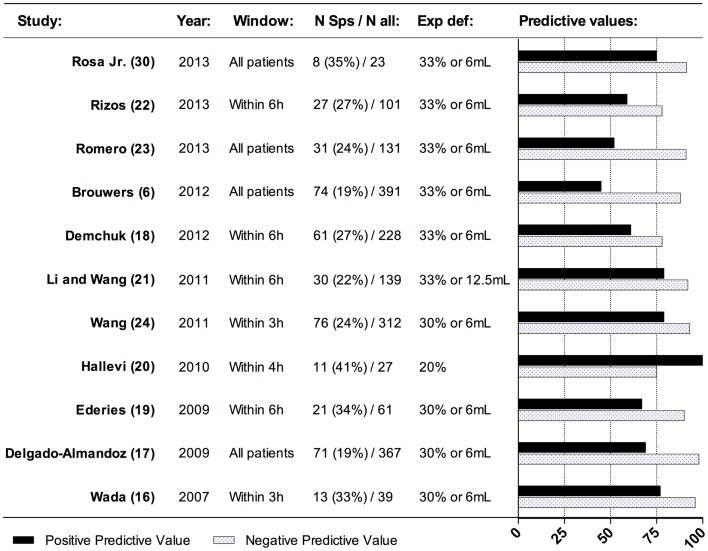
**Effect of spot sign on hematoma expansion**. This figure presents the predictive values across studies for spot sign in predicting hematoma expansion. N Sps, number of patients with spot sign; N all, total number of patients in the population; exp def, definition of hematoma expansion.

The overall prevalence of the spot sign varies among studies from 19 to 41% (6, 16–24) (Figure 4). This reflects the differences in the definition used, different scan protocols and the time-window utilized. In patients arriving within 3 h, the prevalence is reported to be as high as 43–60% (6, 17) dropping down to 11% in patients presenting beyond 6 h after onset (6). This is in good accordance with the proposed temporal profile of post-admission hematoma expansion, because it indicates that the majority of expansion happens within the first hours. The spot sign has been shown to be associated with larger hematoma admission volume (17, 18, 22, 25), higher admission National Institute of Health Stroke Scale (NIHSS) value (18), and hypertension on admission (17, 22). Whether use of vitamin-K antagonists and antiplatelet medication is associated with a higher prevalence of the spot sign, varies between study-results (17, 18, 22). It has been observed that the contrast-phase, in which CTA images were acquired, plays a role in the prevalence of the spot sign. The prevalence of the spot sign was highest in the late venous phase. This might reflect the time required for contrast to accumulate in the hematoma (26).

Across studies, the inter-observer agreement for identifying the spot sign is in general reported to range between good and moderate (κ = 0.60–0.85) (16, 21, 22). In the PREDICT-study, it was observed that even though no definition for the spot sign was selected at the beginning of the study, the agreement in identifying the spot sign between site-investigators and the final expert-adjudication was substantial (κ = 0.72) (18). Huynh et al. (27) presented a study regarding the accuracy of 131 readers to recognize the spot sign, and it was found that physicians in general showed a good specificity (96%), however, the sensitivity was smaller (78%). This might be due to incorrect perception of spot sign mimic. The study further shows that the identification of spot sign proceeded quickly (27). The spot sign must consequently be seen as a relatively easily recognizable biomarker. However, physicians’ ability to recognize it can be improved by simple learning programs (28). This adds greatly to the every-day clinical usefulness of the spot sign in acute ICH-patients.

The spot sign has been proven to be a valid biomarker of hematoma expansion, possibly because of its representation of foci of active bleeding (29). The spot sign in general yields a relatively high negative predictive value (NPV: 0.75–0.98) along with slightly lower positive predictive value (PPV: 0.45–1.00) toward hematoma expansion (Figure 4) (6, 16–24, 30). Sensitivity and specificity are reported to be 0.46–0.91 and 0.84–1.00, respectively (6, 16–24). This is true, even though studies report patients included in different time intervals after ictus. Brouwers et al. (6) found that the positive and negative predictive value remained relatively constant in patients admitted 0–3, 3–6, and >6 h after symptom onset. This indicates the overall ability of the spot sign to predict hematoma expansion across various time-windows. Differences in imaging protocols might also affect the ability of the spot sign to predict hematoma expansion. Spot sign observed in the peak arterial contrast-phase might entail a larger absolute hematoma expansion volume compared to spot sign observed in later venous phases (26). The effect of the spot sign on hematoma expansion is observed to be independent from other predictors of hematoma expansion – especially vitamin-K antagonist use and hematoma volume (16–18, 22) even through the factors might complement each other. In a recently proposed prognostic score, time to initial CT-scan, initial hematoma-size, CTA spot sign, and pre-stroke warfarin treatment were included in order to enable a better stratification of patients with regard to the likelihood of post-admission hematoma expansion (31).

Different amendments to the spot sign have been proposed to increase its ability to identify patients prone to hematoma expansion. Besides the spot sign observed on CTA source images, delayed post-contrast sequences have been utilized to visualize contrast extravasation. Two small studies indicate that the presence of both spot sign and post-contrast extravasation increases the likelihood of hematoma expansion compared to the spot sign alone (19, 20). Another related add-on to the spot sign is the so-called tail-sign (32). The tail-sign is observed originating from the M1 segment of the middle cerebral artery on coronal CTA images in patients with a basal-ganglia hemorrhage and is thought to represent active bleeding from the striate arteries. Only a single smaller study has elaborated the tail-sign, and additional work is needed to confirm its validity.

### Spot sign score

The spot sign score (SSS) was originally proposed by Delgado Almandoz et al. (17) in an attempt to systematically characterize the spot sign and develop a scale capable of predicting hematoma expansion better than the spot sign *per se* was able to. The resulting SSS is presented in Table 1. In the original study, the score provided excellent discriminative capability, when it comes to hematoma expansion (AUC-ROC 0.93 CI: 0.89–0.95), and the risk of hematoma expansion (>30% or 6 mL) increased from 2% in the group with a SSS of 0 to 100% in patients with a SSS of four. Patients with spot sign on admission that arrived in hospital earlier in general presented with a higher SSS compared to patients, who arrived later. The authors later demonstrated that the SSS independently predicts in-hospital mortality and 3 months outcome in an almost linear manner (when increasing from SSS 0 to 4, in-hospital mortality increased from 24 to 75% – and mean 90 days mRS from 3.2 to mRS 5.4) (33).

**Table 1:** **Spot sign score**.

Spot sign characteristics	Points
Number of spot signs
1–2	1
≥3	2
Maximal axial dimension
1–4 mm	0
≥5 mm	1
Maximum attenuation
120–179 HU	0
≥180 HU	1

*Replicated from Delgado Almandoz et al. (17)*.

Other studies have confirmed the independent capability of SSS to predict hematoma expansion (21, 23, 34), mortality, and poor outcome (21, 23) along with the almost linear increase in risk of poor functional outcome with increasing SSS (23). However, not all studies have confirmed that SSS contained superior predictive capabilities of discriminating patients prone to hematoma expansion and poor outcome compared to the spot sign *per se* (21, 34). This might suggest that much of the predictive capability of the SSS is contained within the demonstration of the spot sign. This was emphasized in a *post hoc* analysis from the PREDICT-study revealing no differences between the discriminative capability toward hematoma expansion of the number of spot signs demonstrated and the SSS (34). It was further demonstrated that the risk of hematoma expansion did not increase significantly with increasing SSS. In contrast to this, the risk of hematoma expansion increased significantly with increasing number of spot signs present. The authors speculate that the reason for these differences is that the attenuation of the spot sign – which was proven to have good discriminative ability, when it comes to hematoma expansion in single center studies – might vary between different CT-scanner manufactures along with differences in contrast administration protocol (34).

All in all, the SSS would – if proven valid – provide a better prediction of hematoma expansion useful in clinical decision-making and clinical trials, but so far, the general validity is questionable. However, due to the relative complexity of obtaining the score compared to the spot sign *per se* or number of spot signs, it might represent a less relevant tool in every-day clinical practice and outside clinical trials.

### Relationship between contrast-leakage and outcome

Both the spot sign and contrast extravasation have been shown to be associated with poor outcome. The predictive values are presented in Table 2 for in-hospital mortality, 90 days mortality and 90 days functional outcome.

**Table 2:** **Effect of contrast-leakage in predicting outcome**.

	Design	Extravasation sign	No. contrast-leakage positive (%)	Total number participating	Time-frame	Outcome	Sensitivity (%)	Specificity (%)	PPV (%)	NPV (%)
**In-hospital mortality**
Becker et al. (36)	Retrospective	Contrast extravasation	52 (46)	113	All patients	Hospital fatality	77	73	63	84
Goldstein et al. (37)	Retrospective	Contrast extravasation	58 (56)	104	All patients	Hospital fatality	73	50	33	85
Wada et al. (16)	Retrospective	Spot sign	13 (33)	39	All patients	Hospital fatality	43	69	23	85
Delgado Almandoz et al. (33)	Retrospective	Spot sign	133 (23)	573	All patients	Hospital fatality	41	85	56	76
Li and Wang et al. (21)	Prospective	Spot sign	30 (22)	139	Within 6 h	Hospital fatality	60	81	20	96
Romero et al. (23)	Prospective	Spot sign	31 (24)	131	All patients	Hospital fatality	68	88	61	91
Rizos et al. (22)	Prospective	Spot sign	27 (27)	101	Within 6 h	Hospital fatality	47	77	26	89
**90-days mortality**
Li and Wang et al. (21)	Prospective	Spot sign	30 (22)	139	Within 6 h	Case fatality	50	82	27	93
Demchuk et al. (18)	Prospective	Spot sign	53 (25)	211	Within 6 h	Case fatality	43	81	43	80
Rizos et al. (22)	Prospective	Spot sign	27 (27)	101	Within 6 h	Case fatality	29	74	26	77
**90-days outcome**
Delgado-Almandoz et al. (33)	Retrospective	Spot sign	133 (23)	573	All patients	mRS > 3^a^	23	89	51	70
Li and Wang et al. (21)	Prospective	Spot sign	30 (22)	139	Within 6 h	mRS > 2	36	94	87	58
Romero et al. (23)	Prospective	Spot sign	31 (24)	131	Within 24 h	mRS > 3^a^	34	94	90	50
Havsteen and Ovesen et al. (38)	Prospective	Spot sign	37 (29)	128	Within 4.5 h	mRS > 4	60	86	68	81

*^a^Based on the surviving patients at 3 months*.

The presence of contrast-leakage on admission is associated with short-term clinical outcome (Table 2). It has been shown that the presence of spot sign on admission CTA is linked to neurological deterioration within the first 24 h after symptom onset (18, 35). Across studies, a clear trend toward high negative predictive values of in-hospital mortality was identified, however, with relatively modest or poor positive predictive values (16, 21–23, 33, 36, 37). All in all, the absence of contrast extravasation or spot sign indicates an overall increased stability of the patient during the acute phase of illness. This might be an important observation, as it appears that the difference in mortality between spot sign positive and negative patients manifests during the initial days after symptom onset. After the initial period, the mortality-rate appears to become even between the two groups (18, 38).

The absence of the spot sign in general is associated with good negative predictive values, when it comes to 3 months mortality (18, 21, 22). The spot sign independently increases the probability of long-term fatality (18, 21, 38) and poor functional outcome 3 months after admission in most studies (21, 23, 33, 38). The patients with spot sign in general present a higher 3-month modified Rankin scale score compared to patients without – indicating poorer functional rehabilitation outcome (18, 38). However, in a recent prospective study, this association could not be replicated (22). This finding could represent local variations in treatment and rehabilitation efforts.

## Non-Contrast Computed Tomography

Non-contrast computed tomography (NC-CT) remains the radiological investigation of choice conducted, when stroke patients are admitted for fibrinolysis work-up. Simple and broadly validated predictors of post-admission hematoma expansion observed on NC-CT are therefore essential, because this can potentially predict clinical instability and poor long-term functional outcome.

### Hematoma-size

For a long period of time, the initial admission hematoma volume has been recognized as a major determinant of outcome in patients with acute ICH. In a classic study elaborated by Broderick et al. (39), it was demonstrated that the 30-day mortality in patients with basal-ganglia hemorrhage and a volume above 60 mL was as high as 93% dropping down to 23% for volumes below 30 mL. A similar pattern was observed for patients with lobar hemorrhages.

Other studies have replicated these findings and added other measurements of outcome to the predictive capability of the initial hematoma volume (e.g., in-hospital mortality (40), functional outcome and mortality (40–44), long-term survival (45)), and initial hematoma volume features in most clinical grading scores concerning ICH-patients (46). Hematoma volume and clinical neurological status score on admission (Glasgow Coma Scale, NIHSS, or Canadian Stroke Scale) in general compete for being among the most powerful predictors of outcome.

In the context of acute demonstration of ICH, the natural questions to be raised are: (1) what volume is the most predictive – the early volume with possible on-going hemorrhage or the final hematoma volume? (2) Is it safe to assume that a patient, who arrives early in the hospital and demonstrates a small hematoma volume, is clinically stable and prone to good outcome? 3) What can the hematoma volume tell us about the expansion-potential of the patient?

In an analysis from the VISTA-database (47) on patients presenting with ICH within 6 h after ictus, it was demonstrated that patients with an initial hematoma volume below 20 mL were significantly less likely to undergo hematoma expansion compared to a hematoma volume above 30 mL (using the 12.5 mL expansion-definition). The observation that smaller hematomas are more stable is supported by data from the Recombinant Activated Factor VII ICH Trial (48, 49) along with other individual publications (50, 51). In addition to a decreased rate of post-admission hematoma expansion, smaller hematomas are in general associated with a lower likelihood of early neurological deterioration – and thus a more stable acute course of illness (35, 47, 52).

In a most interesting study elaborated by Rodriguez-Luna et al. (35), the hematoma volume was divided by the time from ictus to admission, yielding an ultra-early hematoma growth pace. It was demonstrated that a higher growth pace was independently associated with the occurrence of post-admission hematoma expansion, early neurological deterioration and poor long-term functional outcome. The discriminative capability of hematoma growth pace was better compared to absolute volume on admission toward early neurological deterioration and poor long-term functional outcome. This method could potentially solve some of the previous proposed uncertainties on the predictive value of hematoma volumes in patients with early presentation due to possible on-going hemorrhage, and the method needs validation in broader populations.

### Intraventricular hemorrhagic extension

Intraventricular hemorrhagic extension is a frequent finding in patients admitted with acute ICH. In patients admitted earlier than 3 h after symptom onset, intraventricular hemorrhagic extension (IVH) is present in 31–48% (22, 35, 38, 53, 54), but delayed IVH can be detected, on follow-up imaging, in up to 20% (53, 54) of the patients without IVH on admission. This is a consequence of post-admission hematoma expansion decompressing into the low resistance ventricular system.

The anatomical location of the intracerebral hematoma is an important determinant for the probability of IVH. Hematomas located in the thalamus or the caudate nucleus will more often extend into the ventricular system due to the proximity (53–57) compared to lobar hematomas. Hallevi et al. (56) calculated for each anatomical position a volume range (decompression range), below which IVH was very unlikely, however, if ICH volume grew above this range, IVH was very likely. This range was very narrow for hematomas located in the thalamus (7.0–12.7 mL) and pons (4.3–11.2 mL) but considerably wider for lobar hematomas (15.4–70.7 mL). This implicates that if a patient with thalamic or pontine hemorrhage without IVH is admitted early after symptom onset or display radiological predictors of active bleeding on admission CT, later re-scan should be considered due to a high probability of delayed IVH. The decompression ranges might also explain the fact that patients with IVH in general present significantly higher admission hematoma volumes compared to patients without (54, 57).

Whether the presence of IVH is associated with hematoma expansion is controversial. Most studies do not find an association between hematoma expansion and IVH, however, one study has shown that lenticular and lobar hematomas with accompanying IVH displayed a higher proportion of hematoma expansion (54). This could be explained by the fact that the expansion-potential is high in a non-periventricular hematoma, if it is able to break through to the ventricles. This finding needs validation in further studies.

Intraventricular hemorrhagic extension may be viewed as a separate hematoma, and expansion of the intraventricular blood volume can occasionally be observed. Steiner et al. (57) reported that 12% of patients with initial IVH suffered enlargement of the IVH volume between baseline and follow-up scan. This expansion was associated with higher baseline ICH hematoma volume, elevated blood-pressure and short time from symptom onset to admission scan. Expansion of the IVH volume was independently associated with poor functional outcome and death.

Intraventricular hemorrhagic extension is reported to be independently associated with both early (<48 h after onset) (10, 35, 58) and late (48 h–1 week after onset) (59) neurological deterioration. There is a high load of evidence indicating that the presence of IVH is independently related to poor clinical outcome and mortality (43, 45, 60–62) mainly due to the obstruction of cerebrospinal fluid flow and hydrocephalus (55, 63), but also by other mechanisms. The association with mortality and poor outcome is likely related to the severity of the IVH volume (57, 61, 64).

### Hematoma heterogenicity (swirl-sign) and irregularity

It is a frequent observation that patients with ICH present hematomas in various shapes and with heterogeneous attenuation on NC-CT. It has for a long period of time been a puzzling hypothesis that this could potentially predict the risk of post-admission hematoma expansion due to the fact that this may signify on-going bleeding.

The observation that a heterogenic attenuation of the hematoma on NC-CT represents on-going bleeding originally descends from observations of patients with epidural hematomas (65). It was observed that the finding of a zone of hypoattenuation within the hyperattenuated hematoma – the so-called “Swirl-sign” – correlated to active bleeding (66). The theory that the swirl-sign could represent on-going bleeding was founded on early studies using computed tomography techniques demonstrating that coagulated and retracted blood-clots appear hyperattenuated compared to normal brain tissue. When blood coagulates, it expels low-density serum leaving behind a high concentration of red blood cells – and thus the globin-protein responsible for the high attenuation (67). Consequently, if a hematoma contains a mixture of coagulated (hyperattenuation) and uncoagulated blood (hypo- or isoattenuation), it appears heterogenic on a NC-CT.

**Figure 5:**
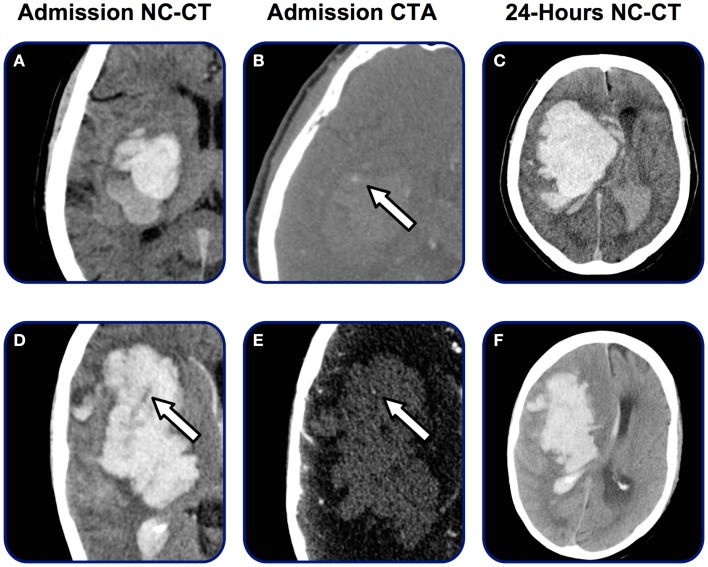
**Heterogenicity on admission NC-CT**. Admission and follow-up radiology on two patients admitted with acute ICH. NC-CT **(A,D)** demonstrates marked heterogenic attenuation in hematoma appearance. Arrow on **(D)** marks swirl-sign. On **(B,E)** both patients present spot sign. After 24 h **(C,F)** both patients present enlargement of the hematoma volume and midline shift.

A number of studies have applied the same principle on patients with acute ICH. Definitions of a heterogeneous attenuation (swirl-sign) (Figure 5) vary between studies, however, a clinically relevant and potentially every-day useful definition proposed by Selariu et al. yielded good intra- and inter-observer agreement: swirl-sign was defined as regions of hypoattenuation or isoattenuation (compared to the attenuation of surrounding brain-parenchyma) within the hyperattenuated ICH (68).

A study elaborated by Barras et al. on patients included on the placebo arm of the Recombinant Activated Factor VII ICH Trial reported that hypoattenuated foci on the admission scan independently predicted some definitions of hematoma expansion, even after adjustment for other predictors of hematoma expansion (48). Additional studies have later confirmed this observation (51, 69). The presence of heterogeneity is further linked to mortality and poor functional outcome (68).

It has been shown that patients arriving in the hospital early have a higher probability of presenting a heterogeneous attenuation of the hematoma. This fits well with the observations of the timing of post-admission hematoma expansion. In one study, the prevalence dropped from 36% in patients arriving within 2 h to 13% in patients arriving later that 24 h after symptom onset (68). The relationship between the swirl-sign and the spot sign would be of pathophysiological and clinical interest and remains to be investigated.

Another proposed marker of active bleeding is irregular shape of the hematoma. The hypothesis behind this observation is that the irregularity could result from multiple leaking vessels feeding the hematoma and hence a higher probability of hematoma expansion (70). Even though the observation that irregularity of the hematoma shape should facilitate hematoma expansion is validated in a large study elaborated by Fujii et al. (71), other studies have not been able to confirm this finding – thus making irregular shaping of the hematoma an uncertain predictor of hematoma expansion (48, 51, 69).

## Follow-Up Imaging in Patients with Intracerebral Hemorrhage

In relation to the substantial amount of patients undergoing post-admission hematoma expansion and the trend toward shorter interval between symptom onset and admission imaging, the need for follow-up imaging after the acute phase of illness becomes more crucial (72). In general, follow-up NC-CT 24 h post-admission is implemented in many centers to evaluate the final hematoma volume, midline shift, the presence of intraventricular or subarachnoid extension of the hemorrhage, and edema formation. NC-CT is in general viewed as the gold standard, when it comes to demonstrating and measuring the volume of ICH. However, CT is limited by the utility of ionizing radiation making it unsuitable for serial assessments of the hematoma volume. In addition, the patients have to leave the stroke wards in most centers in order to undergo CT-studies.

**Figure 6:**
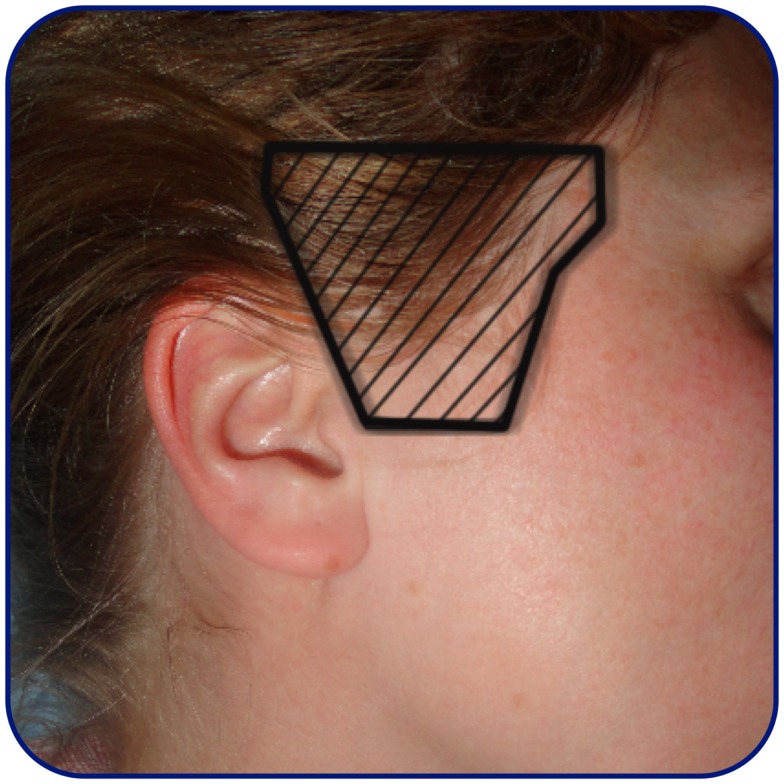
**Trans-temporal ultrasound window**. Approximate location of the trans-temporal ultrasound window.

**Figure 7:**
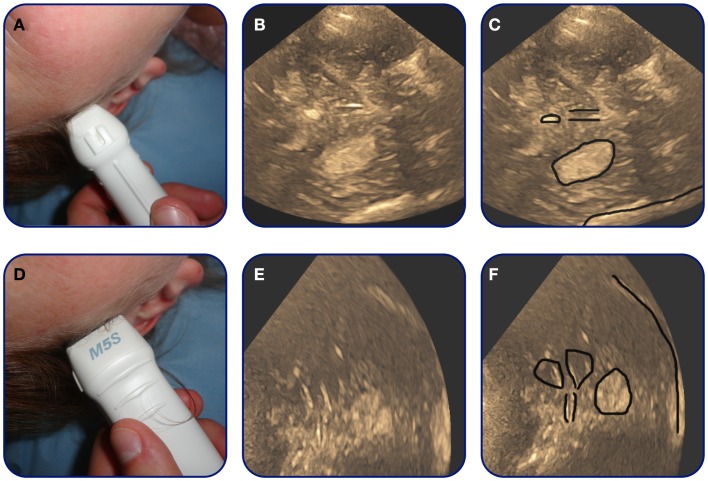
**Transcranial duplex sonography-images of basal-ganglia hemorrhage**. A patient with basal-ganglia ICH. On axial images **(A,B)** the hematoma can be observed as a hyperechogenic region in the middle of the picture. The rim of the hematoma is marked by black on **(C)**. In addition on **(C)** the location of important midline landmark structures as the pineal gland and the third ventricle are marked. **(D,E)** Are coronal images. On **(F)** the lateral ventricles, the third ventricle, and the hematoma is marked.

In recent years, the concept of transcranial duplex sonography (TCDS) has become available to dynamically follow the progression of the intracerebral hematoma on B-mode ultrasound images. The examination is performed through the trans-temporal bone window (Figure 6). On B-mode images, the hematoma is visualized as a hyperechogenic structure compared to surrounding brain-parenchyma. The hematoma is often visualized through the contralateral bone window in reference to the hematoma location (73) (Figure 7). The volume of the hematoma is obtained by measuring the sagittal, transversal, and coronal diameter of the hematoma and then calculating the volume using the standard formula: ABC/2 (74, 75) (Figure 7). Studies indicate that TCDS is able to identify ICH with good sensitivity (73, 75–79). TCDS is also able to estimate the diameters of the hematoma with systematic deviation close to zero (75, 76) and good volume estimation compared to CT (75–77). Studies further indicate that TCDS is useful in following hematoma expansion (75) and demonstrating the presence of IVH (73, 77). In patients with hematomas that are difficult to visualize on TCDS, the administration of ultrasound contrast might improve precision of the estimated volume (78).

Midline shift can also dynamically be followed using TCDS by measuring the distance from the transducer to the third ventricle bilaterally (77, 80, 81). The third ventricle is normally easily recognized on TCDS (Figure 7). By adding Doppler flow studies of the intracranial vessels, TCDS might also be able to estimate intracranial pressure (82, 83), however, additional work needs to examine this in greater detail.

The major limitation to TCDS in patients with ICH is that not all patients present with trans-temporal acoustical bone windows sufficient to allow penetration of diagnostic ultrasound waves. The proportion of the study populations without an acoustical window varies from 7 to 56% in studies on ICH-patients, even though most studies report approximately 20% failure-rate (73, 75, 76, 78, 81, 83). The most notorious predictors of lack of an acoustical window are gender (women in general present poorer acoustical windows), increasing age, and thickness of scull (84, 85). In addition, people of Asian descent in general present with a higher prevalence of inadequate trans-temporal acoustical bone windows compared to Caucasians (76, 84). TCDS is further limited by the anatomical location of the hematoma. Volume estimation of hematomas located either high cortical or infratentorial can be troublesome due to its position in relation to the trans-temporal bone window (73, 75, 77).

Even with the presented limitations, evidence suggests the usefulness of TCDS in patients with ICH as an add-on to the consecutive clinical surveillance during the acute phase of illness.

## Conclusion

Post-admission hematoma expansion in patients with ICH contributes to the clinical deterioration, but may also contain an attractive target for early intervention. CTA for the purpose of evaluating underlying vascular pathology and presence or absence of the spot sign should be considered in all acute ICH-patients. The spot sign has been shown to be a promising biomarker of post-admission hematoma expansion with good predictive capability. In patients with contraindications to CTA predictors of on-going hemorrhage are available for NC-CT. The presence of spot sign, swirl-sign, IVH, or a large hematoma on admission scan marks the patient as potentially clinical unstable and as subject for intensified clinical observation and follow-up imaging. Predictors of hematoma expansion also contain prognostic information toward long-term functional outcome and mortality. TCDS provides a new method to consecutively monitor the development of the hematoma bedside. On-going and future studies will elucidate, if selection of patients for hemostatic therapy based on radiological predictors of post-admission hematoma expansion will finally provide us with an effective treatment-offer for patients with ICH.

## Conflict of Interest Statement

The authors declare that the research was conducted in the absence of any commercial or financial relationships that could be construed as a potential conflict of interest.

## References

[1] Mendelow AD Gregson BA Rowan EN Murray GD Gholkar A Mitchell PM Early surgery versus initial conservative treatment in patients with spontaneous supratentorial lobar intracerebral haematomas (STICH II): a randomised trial. Lancet (2013) 382:397–408 10.1016/S0140-6736(13)60986-1 23726393 PMC3906609

[2] Mayer SA Brun NC Begtrup K Broderick J Davis S Diringer MN Efficacy and safety of recombinant activated factor VII for acute intracerebral hemorrhage. N Engl J Med (2008) 358:2127–37 10.1056/NEJMoa0707534 18480205

[3] Dowlatshahi D Demchuk AM Flaherty ML Ali M Lyden PL Smith EE Defining hematoma expansion in intracerebral hemorrhage: relationship with patient outcomes. Neurology (2011) 76:1238–44 10.1212/WNL.0b013e3182143317 21346218 PMC3068004

[4] Brott T Broderick J Kothari R Barsan W Tomsick T Sauerbeck L Early hemorrhage growth in patients with intracerebral hemorrhage. Stroke (1997) 28:1–5 10.1161/01.STR.28.1.1 8996478

[5] Ovesen C Christensen AF Krieger DW Rosenbaum S Havsteen I Christensen H Time course of early postadmission hematoma expansion in spontaneous intracerebral hemorrhage. Stroke (2014) 45:994–9 10.1161/STROKEAHA.113.003608 24627116

[6] Brouwers HB Falcone GJ McNamara KA Ayres AM Oleinik A Schwab K CTA spot sign predicts hematoma expansion in patients with delayed presentation after intracerebral hemorrhage. Neurocrit Care (2012) 17:421–8 10.1007/s12028-012-9765-2 22878870 PMC3707619

[7] Kazui S Naritomi H Yamamoto H Sawada T Yamaguchi T Enlargement of spontaneous intracerebral hemorrhage. Incidence and time course. Stroke (1996) 27:1783–7 10.1161/01.STR.27.10.1783 8841330

[8] Fisher CM Pathological observations in hypertensive cerebral hemorrhage. J Neuropathol Exp Neurol (1971) 30:536–50 10.1097/00005072-197107000-00015 4105427

[9] Boulouis G Dumas A Betensky RA Brouwers HB Fotiadis P Vashkevich A Anatomic pattern of intracerebral hemorrhage expansion: relation to CT angiography spot sign and hematoma center. Stroke (2014) 45:1154–6 10.1161/STROKEAHA.114.004844 24603066 PMC3984947

[10] Leira R Davalos A Silva Y Gil-Peralta A Tejada J Garcia M Early neurologic deterioration in intracerebral hemorrhage: predictors and associated factors. Neurology (2004) 63:461–7 10.1212/01.WNL.0000133204.81153.AC 15304576

[11] Davis SM Broderick J Hennerici M Brun NC Diringer MN Mayer SA Hematoma growth is a determinant of mortality and poor outcome after intracerebral hemorrhage. Neurology (2006) 66:1175–81 10.1212/01.wnl.0000208408.98482.99 16636233

[12] Delcourt C Huang Y Arima H Chalmers J Davis SM Heeley EL Hematoma growth and outcomes in intracerebral hemorrhage: the INTERACT1 study. Neurology (2012) 79:314–9 10.1212/WNL.0b013e318260cbba 22744655

[13] Yamaguchi K Uemura K Takahashi H Kowada M Kutsuzawa T Intracerebral leakage of contrast medium in apoplexy. Br J Radiol (1971) 44:689–91 10.1259/0007-1285-44-525-689 5569964

[14] Murai Y Takagi R Ikeda Y Yamamoto Y Teramoto A Three-dimensional computerized tomography angiography in patients with hyperacute intracerebral hemorrhage. J Neurosurg (1999) 91:424–31 10.3171/jns.1999.91.3.0424 10470817

[15] Brouwers HB Goldstein JN Romero JM Rosand J Clinical applications of the computed tomography angiography spot sign in acute intracerebral hemorrhage: a review. Stroke (2012) 43:3427–32 10.1161/STROKEAHA.112.664003 23132779 PMC3508322

[16] Wada R Aviv RI Fox AJ Sahlas DJ Gladstone DJ Tomlinson G CT angiography “spot sign” predicts hematoma expansion in acute intracerebral hemorrhage. Stroke (2007) 38:1257–62 10.1161/01.STR.0000259633.59404.f3 17322083

[17] Delgado Almandoz JE Yoo AJ Stone MJ Schaefer PW Goldstein JN Rosand J Systematic characterization of the computed tomography angiography spot sign in primary intracerebral hemorrhage identifies patients at highest risk for hematoma expansion: the spot sign score. Stroke (2009) 40:2994–3000 10.1161/STROKEAHA.109.554667 19574553 PMC3498504

[18] Demchuk AM Dowlatshahi D Rodriguez-Luna D Molina CA Blas YS Dzialowski I Prediction of haematoma growth and outcome in patients with intracerebral haemorrhage using the CT-angiography spot sign (PREDICT): a prospective observational study. Lancet Neurol (2012) 11:307–14 10.1016/S1474-4422(12)70038-8 22405630

[19] Ederies A Demchuk A Chia T Gladstone DJ Dowlatshahi D Bendavit G Postcontrast CT extravasation is associated with hematoma expansion in CTA spot negative patients. Stroke (2009) 40:1672–6 10.1161/STROKEAHA.108.541201 19286577

[20] Hallevi H Abraham AT Barreto AD Grotta JC Savitz SI The spot sign in intracerebral hemorrhage: the importance of looking for contrast extravasation. Cerebrovasc Dis (2010) 29:217–20 10.1159/000267842 20029193

[21] Li N Wang Y Wang W Ma L Xue J Weissenborn K Contrast extravasation on computed tomography angiography predicts clinical outcome in primary intracerebral hemorrhage: a prospective study of 139 cases. Stroke (2011) 42:3441–6 10.1161/STROKEAHA.111.623405 21980207

[22] Rizos T Dorner N Jenetzky E Sykora M Mundiyanapurath S Horstmann S Spot signs in intracerebral hemorrhage: useful for identifying patients at risk for hematoma enlargement? Cerebrovasc Dis (2013) 35:582–9 10.1159/000348851 23859836

[23] Romero JM Brouwers HB Lu J Delgado-Almondoz J Kelly H Heit J Prospective validation of the computed tomographic angiography spot sign score for intracerebral hemorrhage. Stroke (2013) 44(11):3097–102 10.1161/STROKEAHA.113.002752 24021687 PMC4187102

[24] Wang YH Fan JY Luo GD Lin T Xie DX Ji FY Hematoma volume affects the accuracy of computed tomographic angiography ‘spot sign’ in predicting hematoma expansion after acute intracerebral hemorrhage. Eur Neurol (2011) 65:150–5 10.1159/000324153 21372573

[25] Radmanesh F Falcone GJ Anderson CD Battey TW Ayres AM Vashkevich A Risk factors for computed tomography angiography spot sign in deep and lobar intracerebral hemorrhage are shared. Stroke (2014) 45:1833–5 10.1161/STROKEAHA.114.005276 24876264 PMC4116606

[26] Rodriguez-Luna D Dowlatshahi D Aviv RI Molina CA Silva Y Dzialowski I Venous phase of computed tomography angiography increases spot sign detection, but intracerebral hemorrhage expansion is greater in spot signs detected in arterial phase. Stroke (2014) 45(3):734–9 10.1161/STROKEAHA.113.003007 24481974

[27] Huynh TJ Flaherty ML Gladstone DJ Broderick JP Demchuk AM Dowlatshahi D Multicenter accuracy and interobserver agreement of spot sign identification in acute intracerebral hemorrhage. Stroke (2014) 45:107–12 10.1161/STROKEAHA.113.002502 24281226

[28] Havsteen I Christensen A Nielsen JK Christensen L Krieger DW Christensen H E-learn computed tomographic angiography: a proposed educational tool for computed tomographic angiography in acute stroke. J Stroke Cerebrovasc Dis (2012) 21:684–8 10.1016/j.jstrokecerebrovasdis.2011.03.001 21482145

[29] Dowlatshahi D Wasserman JK Momoli F Petrcich W Stotts G Hogan M Evolution of computed tomography angiography spot sign is consistent with a site of active hemorrhage in acute intracerebral hemorrhage. Stroke (2014) 45:277–80 10.1161/STROKEAHA.113.003387 24178918

[30] Rosa Junior M Rocha AJ Saade N Maia Junior AC Gagliardi RJ Active extravasation of contrast within the hemorrhage (spot sign): a multidetector computed tomography finding that predicts growth and a worse prognosis in non-traumatic intracerebral hemorrhage. Arq Neuropsiquiatr (2013) 71:791–7 10.1590/0004-282X20130124 24212517

[31] Brouwers HB Chang Y Falcone GJ Cai X Ayres AM Battey TW Predicting hematoma expansion after primary intracerebral hemorrhage. JAMA Neurol (2014) 71:158–64 10.1001/jamaneurol.2013.5433 24366060 PMC4131760

[32] Sorimachi T Osada T Baba T Inoue G Atsumi H Ishizaka H The striate artery, hematoma, and spot sign on coronal images of computed tomography angiography in putaminal intracerebral hemorrhage. Stroke (2013) 44:1830–2 10.1161/STROKEAHA.113.001498 23674525

[33] Delgado Almandoz JE Yoo AJ Stone MJ Schaefer PW Oleinik A Brouwers HB The spot sign score in primary intracerebral hemorrhage identifies patients at highest risk of in-hospital mortality and poor outcome among survivors. Stroke (2010) 41:54–60 10.1161/STROKEAHA.109.565382 19910545 PMC4181338

[34] Huynh TJ Demchuk AM Dowlatshahi D Gladstone DJ Krischek O Kiss A Spot sign number is the most important spot sign characteristic for predicting hematoma expansion using first-pass computed tomography angiography: analysis from the PREDICT study. Stroke (2013) 44:972–7 10.1161/STROKEAHA.111.000410 23444309

[35] Rodriguez-Luna D Rubiera M Ribo M Coscojuela P Pineiro S Pagola J Ultraearly hematoma growth predicts poor outcome after acute intracerebral hemorrhage. Neurology (2011) 77:1599–604 10.1212/WNL.0b013e3182343387 21998314

[36] Becker KJ Baxter AB Bybee HM Tirschwell DL Abouelsaad T Cohen WA Extravasation of radiographic contrast is an independent predictor of death in primary intracerebral hemorrhage. Stroke (1999) 30:2025–32 10.1161/01.STR.30.10.2025 10512902

[37] Goldstein JN Fazen LE Snider R Schwab K Greenberg SM Smith EE Contrast extravasation on CT angiography predicts hematoma expansion in intracerebral hemorrhage. Neurology (2007) 68:889–94 10.1212/01.wnl.0000257087.22852.21 17372123

[38] Havsteen I Ovesen C Christensen AF Hansen CK Nielsen JK Christensen H Showing no spot sign is a strong predictor of independent living after intracerebral haemorrhage. Cerebrovasc Dis (2014) 37:164–70 10.1159/000357397 24525481

[39] Broderick JP Brott TG Duldner JE Tomsick T Huster G Volume of intracerebral hemorrhage. A powerful and easy-to-use predictor of 30-day mortality. Stroke (1993) 24:987–93 10.1161/01.STR.24.7.987 8322400

[40] Ruiz-Sandoval JL Chiquete E Romero-Vargas S Padilla-Martinez JJ Gonzalez-Cornejo S Grading scale for prediction of outcome in primary intracerebral hemorrhages. Stroke (2007) 38:1641–4 10.1161/STROKEAHA.106.478222 17379820

[41] Cheung RT Zou LY Use of the original, modified, or new intracerebral hemorrhage score to predict mortality and morbidity after intracerebral hemorrhage. Stroke (2003) 34:1717–22 10.1161/01.STR.0000078657.22835.B9 12805488

[42] Godoy DA Pinero G Di Napoli M Predicting mortality in spontaneous intracerebral hemorrhage: can modification to original score improve the prediction? Stroke (2006) 37:1038–44 10.1161/01.STR.0000206441.79646.49 16514104

[43] Hemphill JC III Bonovich DC Besmertis L Manley GT Johnston SC The ICH score: a simple, reliable grading scale for intracerebral hemorrhage. Stroke (2001) 32:891–7 10.1161/01.STR.32.4.891 11283388

[44] Nilsson OG Lindgren A Brandt L Saveland H Prediction of death in patients with primary intracerebral hemorrhage: a prospective study of a defined population. J Neurosurg (2002) 97:531–6 10.3171/jns.2002.97.3.0531 12296635

[45] Zia E Engstrom G Svensson PJ Norrving B Pessah-Rasmussen H Three-year survival and stroke recurrence rates in patients with primary intracerebral hemorrhage. Stroke (2009) 40:3567–73 10.1161/STROKEAHA.109.556324 19729603

[46] Hwang BY Appelboom G Kellner CP Carpenter AM Kellner MA Gigante PR Clinical grading scales in intracerebral hemorrhage. Neurocrit Care (2010) 13:141–51 10.1007/s12028-010-9382-x 20490715

[47] Dowlatshahi D Smith EE Flaherty ML Ali M Lyden P Demchuk AM Small intracerebral haemorrhages are associated with less haematoma expansion and better outcomes. Int J Stroke (2011) 6:201–6 10.1111/j.1747-4949.2010.00563.x 21557804

[48] Barras CD Tress BM Christensen S MacGregor L Collins M Desmond PM Density and shape as CT predictors of intracerebral hemorrhage growth. Stroke (2009) 40:1325–31 10.1161/STROKEAHA.108.536888 19286590

[49] Broderick JP Diringer MN Hill MD Brun NC Mayer SA Steiner T Determinants of intracerebral hemorrhage growth: an exploratory analysis. Stroke (2007) 38:1072–5 10.1161/01.STR.0000258078.35316.30 17290026

[50] Kazui S Minematsu K Yamamoto H Sawada T Yamaguchi T Predisposing factors to enlargement of spontaneous intracerebral hematoma. Stroke (1997) 28:2370–5 10.1161/01.STR.28.12.2370 9412616

[51] Takeda R Ogura T Ooigawa H Fushihara G Yoshikawa S Okada D A practical prediction model for early hematoma expansion in spontaneous deep ganglionic intracerebral hemorrhage. Clin Neurol Neurosurg (2013) 115:1028–31 10.1016/j.clineuro.2012.10.016 23245855

[52] Sorimachi T Fujii Y Early neurological change in patients with spontaneous supratentorial intracerebral hemorrhage. J Clin Neurosci (2010) 17:1367–71 10.1016/j.jocn.2010.02.024 20692165

[53] Maas MB Nemeth AJ Rosenberg NF Kosteva AR Prabhakaran S Naidech AM Delayed intraventricular hemorrhage is common and worsens outcomes in intracerebral hemorrhage. Neurology (2013) 80:1295–9 10.1212/WNL.0b013e31828ab2a7 23516315 PMC3656461

[54] Moussouttas M Malhotra R Fernandez L Maltenfort M Holowecki M Delgado J Impact of intraventricular hemorrhage upon intracerebral hematoma expansion. Neurocrit Care (2011) 14:50–4 10.1007/s12028-010-9452-0 20882367

[55] Bhattathiri PS Gregson B Prasad KS Mendelow AD Intraventricular hemorrhage and hydrocephalus after spontaneous intracerebral hemorrhage: results from the STICH trial. Acta Neurochir Suppl (2006) 96:65–8 10.1007/3-211-30714-1_16 16671427

[56] Hallevi H Albright KC Aronowski J Barreto AD Martin-Schild S Khaja AM Intraventricular hemorrhage: anatomic relationships and clinical implications. Neurology (2008) 70:848–52 10.1212/01.wnl.0000304930.47751.75 18332342 PMC2745649

[57] Steiner T Diringer MN Schneider D Mayer SA Begtrup K Broderick J Dynamics of intraventricular hemorrhage in patients with spontaneous intracerebral hemorrhage: risk factors, clinical impact, and effect of hemostatic therapy with recombinant activated factor VII. Neurosurgery (2006) 59:767–73; discussion 73–4. 10.1227/01.NEU.0000232837.34992.32 17038942

[58] Fan JS Huang HH Chen YC Yen DH Kao WF Huang MS Emergency department neurologic deterioration in patients with spontaneous intracerebral hemorrhage: incidence, predictors, and prognostic significance. Acad Emerg Med (2012) 19:133–8 10.1111/j.1553-2712.2011.01285.x 22320363

[59] Sun W Pan W Kranz PG Hailey CE Williamson RA Sun W Predictors of late neurological deterioration after spontaneous intracerebral hemorrhage. Neurocrit Care (2013) 19:299–305 10.1007/s12028-013-9894-2 23979796 PMC4100944

[60] Hanley DF Intraventricular hemorrhage: severity factor and treatment target in spontaneous intracerebral hemorrhage. Stroke (2009) 40:1533–8 10.1161/STROKEAHA.108.535419 19246695 PMC2744212

[61] Herrick DB Ullman N Nekoovaght-Tak S Hanley DF Awad I Ledroux S Determinants of external ventricular drain placement and associated outcomes in patients with spontaneous intraventricular hemorrhage. Neurocrit Care (2014). 10.1007/s12028-014-9959-x 24522761

[62] Poon MT Fonville AF Al-Shahi Salman R Long-term prognosis after intracerebral haemorrhage: systematic review and meta-analysis. J Neurol Neurosurg Psychiatry (2014) 85:660–7 10.1136/jnnp-2013-306476 24262916

[63] Phan TG Koh M Vierkant RA Wijdicks EF Hydrocephalus is a determinant of early mortality in putaminal hemorrhage. Stroke (2000) 31:2157–62 10.1161/01.STR.31.9.2157 10978045

[64] Tuhrim S Horowitz DR Sacher M Godbold JH Volume of ventricular blood is an important determinant of outcome in supratentorial intracerebral hemorrhage. Crit Care Med (1999) 27:617–21 10.1097/00003246-199903000-00045 10199544

[65] Zimmerman RA Bilaniuk LT Computed tomographic staging of traumatic epidural bleeding. Radiology (1982) 144:809–12 10.1148/radiology.144.4.7111729 7111729

[66] Greenberg J Cohen WA Cooper PR The “hyperacute” extraaxial intracranial hematoma: computed tomographic findings and clinical significance. Neurosurgery (1985) 17:48–56 10.1227/00006123-198507000-00008 4022287

[67] New PF Aronow S Attenuation measurements of whole blood and blood fractions in computed tomography. Radiology (1976) 121:635–40 10.1148/121.3.635 981659

[68] Selariu E Zia E Brizzi M Abul-Kasim K Swirl sign in intracerebral haemorrhage: definition, prevalence, reliability and prognostic value. BMC Neurol (2012) 12:109 10.1186/1471-2377-12-109 23013418 PMC3517489

[69] Ji N Lu JJ Zhao YL Wang S Zhao JZ Imaging and clinical prognostic indicators for early hematoma enlargement after spontaneous intracerebral hemorrhage. Neurol Res (2009) 31:362–6 10.1179/174313209X444035 19508819

[70] Fujii Y Tanaka R Takeuchi S Koike T Minakawa T Sasaki O Hematoma enlargement in spontaneous intracerebral hemorrhage. J Neurosurg (1994) 80:51–7 10.3171/jns.1994.80.1.0051 8271022

[71] Fujii Y Takeuchi S Sasaki O Minakawa T Tanaka R Multivariate analysis of predictors of hematoma enlargement in spontaneous intracerebral hemorrhage. Stroke (1998) 29:1160–6 10.1161/01.STR.29.6.1160 9626289

[72] Maas MB Rosenberg NF Kosteva AR Bauer RM Guth JC Liotta EM Surveillance neuroimaging and neurologic examinations affect care for intracerebral hemorrhage. Neurology (2013) 81:107–12 10.1212/WNL.0b013e31829a33e4 23739227 PMC3770177

[73] Seidel G Kaps M Dorndorf W Transcranial color-coded duplex sonography of intracerebral hematomas in adults. Stroke (1993) 24:1519–27 10.1161/01.STR.24.10.1519 8378956

[74] Kothari RU Brott T Broderick JP Barsan WG Sauerbeck LR Zuccarello M The ABCs of measuring intracerebral hemorrhage volumes. Stroke (1996) 27:1304–5 10.1161/01.STR.27.8.1304 8711791

[75] Perez ES Delgado-Mederos R Rubiera M Delgado P Ribo M Maisterra O Transcranial duplex sonography for monitoring hyperacute intracerebral hemorrhage. Stroke (2009) 40:987–90 10.1161/STROKEAHA.108.524249 19164795

[76] Matsumoto N Kimura K Iguchi Y Aoki J Evaluation of cerebral hemorrhage volume using transcranial color-coded duplex sonography. J Neuroimaging (2011) 21:355–8 10.1111/j.1552-6569.2010.00559.x 21143547

[77] Kukulska-Pawluczuk B Ksiazkiewicz B Nowaczewska M Imaging of spontaneous intracerebral hemorrhages by means of transcranial color-coded sonography. Eur J Radiol (2012) 81:1253–8 10.1016/j.ejrad.2011.02.066 21435810

[78] Kern R Kablau M Sallustio F Fatar M Stroick M Hennerici MG Improved detection of intracerebral hemorrhage with transcranial ultrasound perfusion imaging. Cerebrovasc Dis (2008) 26:277–83 10.1159/000147456 18648201

[79] Maurer M Shambal S Berg D Woydt M Hofmann E Georgiadis D Differentiation between intracerebral hemorrhage and ischemic stroke by transcranial color-coded duplex-sonography. Stroke (1998) 29:2563–7 10.1161/01.STR.29.12.2563 9836768

[80] Kiphuth IC Huttner HB Breuer L Schwab S Kohrmann M Sonographic monitoring of midline shift predicts outcome after intracerebral hemorrhage. Cerebrovasc Dis (2012) 34:297–304 10.1159/000343224 23146822

[81] Tang SC Huang SJ Jeng JS Yip PK Third ventricle midline shift due to spontaneous supratentorial intracerebral hemorrhage evaluated by transcranial color-coded sonography. J Ultrasound Med (2006) 25:203–9 16439783 10.7863/jum.2006.25.2.203

[82] Kiphuth IC Huttner HB Dorfler A Schwab S Kohrmann M Doppler pulsatility index in spontaneous intracerebral hemorrhage. Eur Neurol (2013) 70:133–8 10.1159/000350815 23887314

[83] Marti-Fabregas J Belvis R Guardia E Cocho D Marti-Vilalta JL Relationship between transcranial Doppler and CT data in acute intracerebral hemorrhage. AJNR Am J Neuroradiol (2005) 26:113–8 15661712 PMC7975030

[84] Kwon JH Kim JS Kang DW Bae KS Kwon SU The thickness and texture of temporal bone in brain CT predict acoustic window failure of transcranial Doppler. J Neuroimaging (2006) 16:347–52 10.1111/j.1552-6569.2006.00064.x 17032385

[85] Wijnhoud AD Franckena M van der Lugt A Koudstaal PJ Dippel ED Inadequate acoustical temporal bone window in patients with a transient ischemic attack or minor stroke: role of skull thickness and bone density. Ultrasound Med Biol (2008) 34:923–9 10.1016/j.ultrasmedbio.2007.11.022 18243493

